# Progress Toward Regional Measles Elimination — Worldwide, 2000–2016

**DOI:** 10.15585/mmwr.mm6642a6

**Published:** 2017-10-27

**Authors:** Alya Dabbagh, Minal K. Patel, Laure Dumolard, Marta Gacic-Dobo, Mick N. Mulders, Jean-Marie Okwo-Bele, Katrina Kretsinger, Mark J. Papania, Paul A. Rota, James L. Goodson

**Affiliations:** ^1^Department of Immunization, Vaccines, and Biologicals, World Health Organization; ^2^Global Immunization Division, Center for Global Health, CDC; ^3^Division of Viral Diseases, National Center for Immunization and Respiratory Diseases, CDC.

The fourth United Nations Millennium Development Goal, adopted in 2000, set a target to reduce child mortality by two thirds by 2015. One indicator of progress toward this target was measles vaccination coverage (*1*). In 2010, the World Health Assembly (WHA) set three milestones for measles control by 2015: 1) increase routine coverage with the first dose of a measles-containing vaccine (MCV1) among children aged 1 year to ≥90% at the national level and to ≥80% in every district; 2) reduce global annual measles incidence to <5 cases per million population; and 3) reduce global measles mortality by 95% from the 2000 estimate (*2*).* In 2012, WHA endorsed the Global Vaccine Action Plan,^†^ with the objective of eliminating measles in four World Health Organization (WHO) regions by 2015 and in five regions by 2020. Countries in all six WHO regions have adopted goals for measles elimination by or before 2020. Measles elimination is defined as the absence of endemic measles virus transmission in a region or other defined geographic area for ≥12 months, in the presence of a high quality surveillance system that meets targets of key performance indicators. This report updates a previous report (*3*) and describes progress toward global measles control milestones and regional measles elimination goals during 2000–2016. During this period, annual reported measles incidence decreased 87%, from 145 to 19 cases per million persons, and annual estimated measles deaths decreased 84%, from 550,100 to 89,780; measles vaccination prevented an estimated 20.4 million deaths. However, the 2015 milestones have not yet been met; only one WHO region has been verified as having eliminated measles. Improved implementation of elimination strategies by countries and their partners is needed, with focus on increasing vaccination coverage through substantial and sustained additional investments in health systems, strengthening surveillance systems, using surveillance data to drive programmatic actions, securing political commitment, and raising the visibility of measles elimination goals.

## Immunization Activities

To estimate coverage with MCV1 and the second dose of measles-containing vaccine (MCV2) through routine immunization services,^§^ WHO and the United Nations Children’s Fund (UNICEF) use data from administrative records (administrative coverage is calculated by dividing the vaccine doses administered by the estimated target population) and immunization coverage surveys reported annually by 194 countries. During 2000–2016, estimated MCV1 coverage increased globally from 72% to 85% (Table 1), although coverage has not increased since 2009. Considerable variability in regional coverage exists. Since 2012, MCV1 coverage has remained essentially unchanged in the African Region (AFR) (72%), the Region of the Americas (AMR) (92%), and the Eastern Mediterranean Region (EMR) (77%). In the European Region (EUR), MCV1 coverage has declined from 95% to 93% since 2012, with 51% of EUR member states reporting lower coverage since 2013. In the South-East Asia Region (SEAR), MCV1 coverage increased slightly since 2012, from 84% to 87%. The Western Pacific Region (WPR) is the only region that has achieved and sustained MCV1 coverage >95% (since 2008). Since 2000, the number of countries with MCV1 coverage of ≥90% increased globally from 85 (44%) in 2000 to 119 (61%) in 2015, and to 123 (63%) in 2016. However, among countries with ≥90% MCV1 coverage nationally, the percentage with ≥80% MCV1 coverage in all districts declined from 46% (52 of 112) in 2010 to 45% (49 of 110) in 2015 and 36% (44 of 123) in 2016. Among the estimated 20.8 million infants who did not receive MCV1 through routine immunization services in 2016, approximately 11 million (53%) were in six countries with large birth cohorts and suboptimal coverage: Nigeria (3.3 million), India (2.9 million), Pakistan (2.0 million), Indonesia (1.2 million), Ethiopia (0.9 million), and the Democratic Republic of the Congo (0.7 million).

**TABLE 1:** Estimates of coverage with the first and second doses of measles-containing vaccine administered through routine immunization services, reported measles cases and incidence, and estimated measles deaths,* by World Health Organization (WHO) region — worldwide, 2000 and 2016

WHO region (no. countries in region)/Year	% Coverage with MCV1^†^	% Countries with ≥90% MCV1 coverage	% Coverage with MCV2^†^	% Countries with incidence <5/million	No. reported measles cases^§^	Measles incidence^§,¶^	Estimated no. of measles deaths (95% CI)	% Estimated mortality reduction, 2000–2016
**African (47)**								
2000	53	9	5	8	520,102	835	340,800 (232,000–554,000)	89
2016	72	36	24	51	36,269	36	37,500 (11,900–124,200)	
**Americas (35)**								
2000	93	63	43	89	1,754	2.1	NA	**—**
2016	92	74	54	100	12	0.02	NA	
**Eastern Mediterranean (21)**								
2000	72	57	29	17	38,592	90	55,300 (35,000–87,700)	79
2016	77	57	69	47	6,264	10	11,400 (5,700–28,300)	
**European (53)**								
2000	91	60	48	45	37,421	50	400 (130–2,000)	80
2016	93	83	88	85	4,175	5	80 (0–1,400)	
**South-East Asia (11)**								
2000	63	30	3	0	78,558	51	143,000 (101,500–199,900)	73
2016	87	64	75	27	27,530	14	39,000 (27,600–69,700)	
**Western Pacific (27)**								
2000	85	48	2	30	177,052	105	10,600 (5,200–52,400)	83
2016	96	63	93	67	57,879	31	1,800 (500–46,000)	
**Total (194)**								
2000	72	44	15	38	853,479	145	550,100 (374,000–896,500)	84
2016	85	63	64	69	132,137	19	89,780 (45,700–269,600)	

**Abbreviations:** CI = confidence interval; MCV1 = first dose of measles-containing vaccine; MCV2 = second dose of measles-containing vaccine; NA = not applicable; UNICEF = United Nations Children’s Fund.

* Mortality estimates for 2000 might be different from previous reports. When the model used to generate estimated measles deaths is rerun each year using the new WHO/UNICEF Estimates of National Immunization Coverage data, as well as updated surveillance data, adjusted results for each year, including the baseline year, are also produced and updated.

^†^ Coverage data: WHO/UNICEF Estimates of National Immunization Coverage, July 15, 2017 update. http://www.who.int/immunization/monitoring_surveillance/data/en.

^§^ Reported case data: measles cases (2016) from World Health Organization, as of July 15, 2017 (http://apps.who.int/immunization_monitoring/globalsummary/timeseries/tsincidencemeasles.html). Reported cases are a sizeable underestimate of the actual number of cases, accounting for the inconsistency between reported cases and estimated deaths.

^¶^ Cases per 1 million population; population data from United Nations, Department of Economic and Social Affairs, Population Division, 2016. Any country not reporting data on measles cases for that year was removed from both the numerator and denominator.

During 2000–2016, the number of countries providing MCV2 nationally through routine services increased from 98 (51%) to 164 (85%), with four countries (Guatemala, Haiti, Papua New Guinea, and Timor-Leste) introducing MCV2 in 2016. Estimated global MCV2 coverage steadily increased from 15% in 2000 to 60% in 2015 and 64% in 2016 (Table 1). During 2016, approximately 119 million persons received supplementary doses of measles-containing vaccine (MCV) during 33 mass immunization campaigns, known as supplementary immunization activities (SIAs),^¶^ implemented in 31 countries (Table 2). Based on doses administered, SIA coverage was ≥95% in 20 (61%) SIAs. Among the six countries that conducted post-SIA coverage surveys, estimated coverage was ≥95% in three, 90%–94% in two, and 84% in one.

**TABLE 2:** Measles supplementary immunization activities (SIAs)* and the delivery of other child health interventions, by World Health Organization (WHO) region and country — worldwide, 2016

WHO region/country	Age group targeted	Extent of SIA	No. children reached in targeted age group (%)^†^	% coverage based on survey results	Other interventions delivered
**African**					
Botswana	9 mos–14 yrs	N	674,150 (95)	97	Rubella vaccine
Burundi (2015–2016)^§^	18–23 mos	N	30,443 (22)	—	—
Central African Republic (2015–2016)^§^	6 mos–10 yrs	N	1,529,441 (84)	—	Vitamin A, deworming
Chad	9–59 mos	N	2,756,733 (110)	—	—
Comoros	9–59 mos	SN	83,371 (76)	—	Vitamin A, deworming
Democratic Republic of the Congo	6–59 mos	N	10,921,820 (100)	—	—
Equatorial Guinea	6–59 mos	N	127,874 (85)	—	—
Ethiopia	6 mos–15 yrs	SN	24,986,589 (97)	94	—
Gambia	9 mos–14 yrs	N	779,654 (97)	97	Rubella vaccine, vitamin A, deworming
Guinea	9–59 mos	N	2,412,923 (103)	—	Vitamin A, deworming
Kenya	9 mos–14 yrs	N	19,154,577 (101)	95	Rubella vaccine
Madagascar	9–59 mos	N	3,547,466 (96)	—	Vitamin A, deworming
Namibia	9 mos–39 yrs	N	1,908,193 (103)	—	Rubella vaccine
Nigeria	9–59 mos	N	19,065,787 (131)	84	—
Sao Tome and Principe	9 mos–14 yrs	N	77,285 (107)	—	Rubella vaccine
Swaziland	9 mos–14 yrs	N	373,508 (90)	94	Rubella vaccine, vitamin A, deworming
Zambia	9 mos–14 yrs	N	7,741,505 (108)	—	Rubella vaccine
**Americas**					
Haiti	9–59 mos	N	1,420,220 (100)	—	Rubella vaccine, OPV, IPV, vitamin A
Honduras	1–4 yrs	N	735,066 (96)	—	Mumps and rubella vaccine
Mexico	1–4 yrs	N	8,229,851 (94)	—	Mumps and rubella vaccine
Nicaragua	1–4 yrs	N	568,422 (105)	—	Mumps and rubella vaccine
Peru	2–5 yrs	N	1,662,728 (78)	—	Rubella vaccine
**Eastern Mediterranean**					
Egypt	11–20 yrs	SN	642,178 (94)	—	Rubella vaccine
Egypt	6–7 yrs (1st grade)	SN	258,464 (102)	—	Rubella vaccine
Qatar	1–13 yrs	N	166,145 (87)	—	Mumps and rubella vaccine
**South-East Asia**					
Bangladesh	9–59 mos	SN	100,863 (101)	—	Rubella vaccine
Indonesia	9–59 mos	SN	3,638,183 (86)	—	
Nepal	9–59 mos	N	2,528,539 (101)	—	Rubella vaccine
**Western Pacific**					
Malaysia	6 m–17 yrs	SN	139,382 (85)	—	Rubella vaccine
Malaysia	1–17 yrs	SN	572 (99)	—	Rubella vaccine
Mongolia	18–30 yrs	N	549,846 (88)	—	Rubella vaccine
Papua New Guinea	9 mos–15 yrs	SN	436,854 (63)	—	Rubella vaccine
Vietnam	16–17 yrs	N	1,787,588 (95)	—	Rubella vaccine

**Abbreviations:** IPV = inactivated polio vaccine; N = National; OPV = oral polio vaccine; SIA = supplementary immunization activity; SN = subnational.

* SIAs generally are carried out using two approaches: 1) An initial, nationwide catch-up SIA targets all children aged 9 months to 14 years; it has the goal of eliminating susceptibility to measles in the general population. Periodic follow-up SIAs then target all children born since the last SIA. 2) Follow-up SIAs are generally conducted nationwide every 2–4 years and target children aged 9–59 months; their goal is to eliminate any measles susceptibility that has developed in recent birth cohorts and to protect children who did not respond to the first measles vaccination. The exact age range for follow-up SIAs depends on the age-specific incidence of measles, coverage with 1 dose of measles-containing vaccine, and the time since the last SIA.

^†^ Values >100% indicate that the intervention reached more persons than the estimated target population.

^§^ Rollover national campaigns started the previous year or will continue into the next year.

## Disease Incidence

Countries report the aggregate number of incident measles cases**^,^^††^ to WHO and UNICEF annually through the Joint Reporting Form. In 2016, 189 (97%) countries conducted case-based surveillance in at least part of the country, and 191 (98%) had access to standardized quality-controlled testing through the WHO Global Measles and Rubella Laboratory Network. Nonetheless, surveillance was weak in many countries; fewer than half of countries (64 of 134; 48%) achieved the sensitivity indicator target of two or more discarded measles and rubella^§§^ cases per 100,000 population in 2016 compared with 2015 (80 of 135; 59%).

During 2000–2016, the number of measles cases reported annually worldwide decreased 85%, from 853,479 in 2000 to 214,812 in 2015 and then to 132,137 in 2016; measles incidence decreased 87%, from 145 to 19 cases per 1 million population (Table 1). Compared with 2015, 2016 incidence decreased from 29 to 19 cases per million, although three fewer countries (173 of 194; 89%) reported case data in 2016 than did in 2015 (176 of 194; 92%).^¶¶^ The percentage of reporting countries with fewer than five measles cases per million population increased from 38% (64/169) in 2000 to 69% (119/173) in 2016. During 2000–2016, measles incidence of fewer than five cases per million was sustained in AMR (Table 1).

During 2015–2016, the number of reported measles cases declined globally and in all regions (AFR, 31%; AMR, 98%; EMR, 71%; EUR, 84%; SEAR, 44%, and WPR, 11%). In addition to aggregate reporting, countries report measles case-based data to WHO monthly. In some countries large discrepancies exist between the two reporting systems. During 2016, some countries either did not report or reported only a fraction of monthly reported measles cases through the Joint Reporting Form (e.g., India reported 70,798 measles cases through monthly reporting, but only 17,250 through the Joint Reporting Form).

Genotypes of viruses isolated from measles cases were reported by 60 (55%) of the 110 countries that reported at least one measles case in 2016. Among the 24 recognized measles virus genotypes, 11 were detected during 2005–2008, eight during 2009–2014, six in 2015, and five in 2016, excluding those from vaccine reactions and cases of subacute sclerosing panencephalitis, a fatal progressive neurologic disorder caused by persistent measles infection (*4*).*** In 2016, among 4,796 reported measles virus sequences,^†††^ 666 were genotype B3 (36 countries); 44 were D4 (four); 1,407 were D8 (43); 87 were D9 (four); and 2,592 were H1 (13).

## Disease and Mortality Estimates

A previously described model for estimating measles disease and mortality was updated with new measles vaccination coverage data, case data, and United Nations population estimates for all countries during 2000–2016, enabling derivation of a new series of disease and mortality estimates (*5*). Based on the updated data, the estimated number of measles cases declined from 29,068,400 (95% confidence interval [CI] = 20,606,800–55,859,000) in 2000 to 6,976,800 (95% CI = 4,190,500–28,657,300) in 2016. During this period, the number of estimated measles deaths declined 84%, from 550,100 (95% CI = 374,000–896,500) in 2000 to 89,780 (95% CI = 45,700–269,600) in 2016 (Table 1). Compared with no measles vaccination, measles vaccination prevented an estimated 20.4 million deaths during 2000–2016 (Figure).

**FIGURE:**
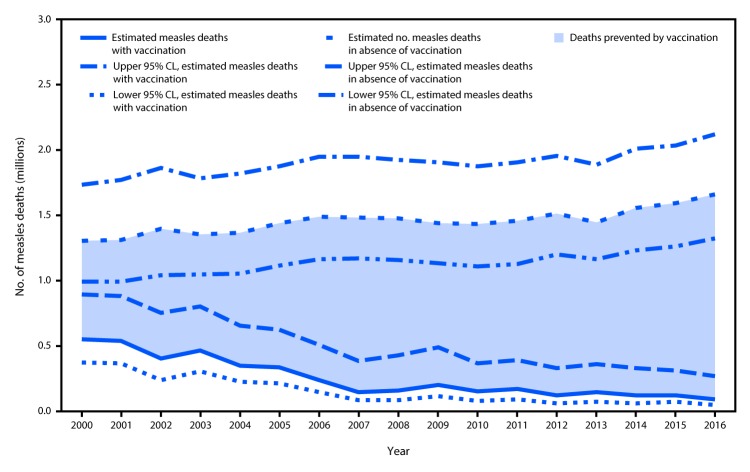
Estimated annual number of measles deaths with and without vaccination programs — worldwide, 2000–2016* **Abbreviation:** CL = confidence limit. * Deaths prevented by vaccination is indicated by the shaded area between estimated deaths with vaccination and those without vaccination (cumulative total of 20.4 million deaths prevented during 2000–2016).

## Regional Verification of Measles Elimination

In 2016, four WHO regions had functioning regional verification commissions. In September 2016, the AMR regional verification commission declared the region free of endemic measles (*6*). In 2016, the EUR commission verified measles elimination in 24 countries (*7*). Two SEAR countries (Bhutan and Maldives) were verified as having eliminated measles in 2017 (*8*). The WPR commission reclassified Mongolia as having reestablished endemic measles virus transmission because of an outbreak that lasted >12 months; thus, five WPR countries (Australia, Brunei, Cambodia, Japan, and South Korea) and two areas (Macao Special Autonomous Region [SAR] [China] and Hong Kong SAR [China]) had verified measles elimination status in 2016 (*9*).

## Discussion

During 2000–2016, increased coverage with MCV administered through routine immunization programs worldwide, combined with SIAs, contributed to an 87% decrease in reported measles incidence and an 84% reduction in estimated measles mortality. Measles vaccination prevented an estimated 20.4 million deaths during this period, and during 2016, for the first time ever, estimated measles deaths declined to fewer than 100,000. Furthermore, the number of countries with measles incidence of fewer than five per million population has increased, although considerable underreporting occurred, and AMR has maintained an incidence of fewer than five cases per million population during 2000–2016. The decreasing number of circulating measles virus genotypes suggests interruption of some chains of transmission. However, the 2015 global control milestones were not met, global MCV1 coverage has stagnated, global MCV2 coverage has reached only 64%, and SIA quality was inadequate to achieve ≥95% coverage in several countries. With suboptimal MCV coverage, outbreaks continued to occur among unvaccinated persons, including school-aged children and young adults.

The 2016 Mid-term Review of the Global Measles and Rubella Strategic Plan 2012–2020 concluded that measles elimination strategies were sound, and the WHO Strategic Advisory Group of Experts on Immunization endorsed its findings. The review noted, however, that implementation of the strategies needs improvement. Measures should focus on strengthening immunization and surveillance systems. The Measles and Rubella Initiative should increase its emphasis on using surveillance data to drive programmatic actions.

The findings in this report are subject to at least three limitations. First, SIA coverage data might be biased by inaccurate reports of the number of doses delivered, doses administered to children outside the target age group, and inaccurate estimates of the target population size. Second, large differences between the estimated and reported incidence indicate variable surveillance sensitivity, making comparisons between countries and regions difficult to interpret. Finally, the accuracy of the results from the measles mortality model is affected by biases in all model inputs, including country-specific measles vaccination coverage and measles case-based surveillance data.

The decrease in measles mortality to fewer than 100,000 deaths in 2016 is one of five main contributors (along with decreases in mortality from diarrhea, malaria, pneumonia, and neonatal intrapartum deaths) to the decline in overall child mortality worldwide and progress toward the fourth United Nations Millennium Development Goal, but continued work is needed to help achieve measles elimination goals (*10*). Of concern is the possibility that the gains made and future progress in measles elimination could be reversed when polio-funded resources supporting routine immunization services, measles SIAs, and measles surveillance diminish and disappear after polio eradication. Countries with the highest measles mortality rely most heavily on polio-funded resources and are at highest risk for reversal of progress after polio eradication is achieved. Improved implementation of elimination strategies by countries and their partners is needed, with focus on increasing vaccination coverage with substantial and sustained additional investments in health systems, strengthening surveillance systems, using surveillance data to drive programmatic actions, securing political commitment, and raising the visibility of measles elimination goals.

**Summary**

**What is already known about this topic?**

The fourth United Nations Millennium Development Goal, adopted in 2000, set a target to reduce child mortality by two thirds by 2015. One indicator of progress toward this target was measles vaccination coverage.

**What is added by this report?**

For the first time, annual estimated measles deaths were fewer than 100,000, in 2016. This achievement follows an increase in the number of countries providing the second dose of measles-containing vaccine (MCV2) nationally through routine immunization services to 164 (85%) of 194 countries, and the vaccination of approximately 119 million persons against measles during supplementary immunization activities in 2016. During 2000–2016, annual reported measles incidence decreased 87%, from 145 to 19 cases per million persons, annual estimated measles deaths decreased 84%, from 550,100 to 89,780, and an estimated 20.4 million deaths were prevented. However, the 2015 measles elimination milestones have not yet been met, and only one World Health Organization region has been verified as having eliminated measles.

**What are the implications for public health practice?**

To achieve measles elimination goals, countries and their partners need to act urgently to secure political commitment, raise the visibility of measles elimination, increase vaccination coverage, strengthen surveillance, and mitigate the threat of decreasing resources once polio eradication is achieved. Polio eradication resources have supported routine immunization services and surveillance activities.

## Footnotes

* The coverage milestone is to be met by every country, whereas the incidence and mortality reduction milestones are to be met globally.

† The Global Vaccine Action Plan is the implementation plan of the Decade of Vaccines, a collaboration between WHO; the United Nations Children’s Fund (UNICEF); the Bill and Melinda Gates Foundation; the National Institute of Allergy and Infectious Diseases; the African Leaders Malaria Alliance; Gavi, the Vaccine Alliance; and others to extend the full benefit of immunization to all persons by 2020 and beyond. In addition to 2015 targets, it also set a target for measles and rubella elimination in five of the six WHO regions by 2020. http://www.who.int/immunization/global_vaccine_action_plan/en; http://apps.who.int/gb/ebwha/pdf_files/wha65/a65_22-en.pdf.

§ For the first dose of measles-containing vaccine (MCV1), among children aged 1 year or, if MCV1 is given at age ≥1 year, among children aged 24 months. For MCV2, among children at the recommended age for administration of MCV2, per the national immunization schedule. WHO/UNICEF estimates of national immunization coverage are available at http://www.who.int/immunization/monitoring_surveillance/data/en.

¶ Supplemental immunization activities (SIAs) generally are carried out using two target age ranges. An initial, nationwide catch-up SIA focuses on all children aged 9 months–14 years, with the goal of eliminating susceptibility to measles in the general population. Periodic follow-up SIAs then focus on all children born since the last SIA. Follow-up SIAs generally are conducted nationwide every 2–4 years and focus on children aged 9–59 months; their goal is to eliminate any measles susceptibility that has developed in recent birth cohorts and to protect children who did not respond to MCV1.

** http://apps.who.int/immunization_monitoring/globalsummary/timeseries/tsincidencemeasles.html.

†† Measles cases are defined differently in different countries. Some countries define measles cases as those that are laboratory-confirmed or epidemiologically linked; others define measles cases as those that are laboratory-confirmed, epidemiologically linked, or clinically compatible. Laboratory-confirmed cases are suspected measles cases with specimens that have detectable measles virus-specific immunoglobulin class M (IgM) antibodies, or specimens from which measles virus can be isolated or measles virus genome can be detected in appropriate clinical specimens by a proficient laboratory. Epidemiologically linked confirmed measles cases are suspected measles cases that have not been confirmed by a laboratory but are geographically and temporally related to a laboratory-confirmed case or, in the event of a chain of transmission, to another epidemiologically confirmed measles case, with dates of rash onset between cases occurring 7–21 days apart. Clinically compatible measles cases are suspected measles cases with fever and maculopapular rash and cough, coryza, or conjunctivitis, for which no adequate clinical specimen was collected and which have not been linked epidemiologically to a laboratory-confirmed case of measles or to a laboratory-confirmed case of another communicable disease.

§§ A discarded case is defined as a suspected case that has been investigated and discarded as nonmeasles and as nonrubella using 1) laboratory testing in a proficient laboratory or 2) epidemiological linkage to a laboratory-confirmed outbreak of a communicable disease that is not measles or rubella. The discarded case rate is used to measure the sensitivity of measles surveillance.

¶¶ Twenty-one countries did not report measles case data in 2016: Antigua and Barbuda, Belgium, Cabo Verde, Cook Islands, Ireland, Italy, Kiribati, Marshall Islands, Monaco, Morocco, Mozambique, Nauru, Niue, Poland, Portugal, Samoa, Singapore, Switzerland, Tuvalu, United States, and Vanuatu.

*** http://dx.doi.org/10.1016/B978-0-444-53488-0.00027-4.

††† Sequences were for the 450 nucleotides coding for the carboxy-terminal 150 amino acids of the nucleoprotein of measles virus. Data (as of September 16, 2017) are available from the Measles Nucleotide Surveillance database. http://www.who-measles.org/Public/Web_Front/main.php.
